# Neurobrucellosis complicated with cerebral venous sinus thrombosis in a young Syrian male: a case report

**DOI:** 10.1097/MS9.0000000000002022

**Published:** 2024-04-03

**Authors:** Ayham Alhusseini, Omar Alsamarrai, Mohammad Alsultan, Nawwar Soliman, Suaad Hamsho

**Affiliations:** a Department of Neurology; b Department of Nephrology; c Department of Internal Medicine; d Department of Rheumatology, Al Assad and Al Mouwasat University Hospitals, Faculty of Medicine, Damascus University, Damascus, Syria

**Keywords:** brucella, cerebral venous sinus thrombosis (CVST), neurobrucellosis

## Abstract

**Introduction and importance:**

Neurobrucellosis occurs when Brucella affects the nervous system and it has several presentations. One of its rarest complications is cerebral venous sinus thrombosis (CVST).

**Case presentation:**

A 16-year-old male patient complaining of a sudden onset of bilateral pulsatile headache accompanied by fever, dizziness, nausea, vomiting, and blurred vision. On neurological examination the patient had neck stiffness and a bilateral 2nd degree papilledema. Brain computed tomography did not reveal any space-occupying lesions. Lumbar puncture showed an elevated lymphocyte count in the CSF and the Brucella PCR was positive. MRI with contrast and magnetic resonance venography revealed a left transverse sinus thrombosis and a diagnosis of neurobrucellosis complicated with CVST was made.

**Discussion:**

CVST is a rare but serious complication of neurobrucellosis, it has been described in only a handful of cases. The diagnosis mainly consists of establishing the presence of neurobrucellosis using the CSF analysis and the Brucella PCR, and proving the existence of CVST using the brain MRI. Although, the management of this complication remains a controversy, the use of an antibiotic combination and anticoagulation therapy may improve the symptoms greatly.

**Conclusion:**

Although Brucella seldom affects the nervous system, with CVST being an extremely rare complication. Physicians should consider brucella as the cause of CVST, in endemic areas. Usually, it is treated with a combination of antibiotics. However, anticoagulation should be considered in some cases and future studies must be conducted to assess the role of anticoagulation treatment.

## Introduction

Highlights

- Brucellosis is the most common zoonotic infection worldwide.
- The most common transmission method of Brucella is consuming unpasteurized milk.
- Symptoms range from mild to severe including fever, malaise, and night sweats are the most common symptoms.
- Cerebral vascular complications of neurobrucellosis, whether they are thrombotic or hemorrhagic, are rare, making the association between neurobrucellosis and cerebral venous sinus thrombosis even more rare.
- Treatment of neurobrucellosis is a combination of 2 or 3 antibiotics and whether the anticoagulation therapy, in neurobrucellosis induced cerebral venous sinus thrombosis, is useful or not remains controversial.

This work has been reported in the line of Surgical CAse REport (SCARE) checklist^1^.

Brucellosis is the most common zoonotic infection worldwide including in the Mediterranean, Middle Eastern countries, Central Asia, China, Mexico, and Central and South America^2^, it affects 500 000 cases annually^3^; causing major health and economic problems in these areas^2^. The most common transmission method for Brucella is consumption of unpasteurized milk, cheese, or butter. In addition, it can be transmitted by direct contact with infected animals, and through the inhalation of infected aerosolized particles^4^. Brucellosis is a multisystem disease. Symptoms range from mild to severe with fever, malaise, and night sweats being the most common^5^.

When Brucella affects the nervous system, it is called neurobrucellosis, which is a relatively rare condition that can occur in about 5–7% of Brucella infections. The most common presentation is meningitis and meningoencephalitis; also, brain abscesses and demyelinating syndromes have been reported^6^. However, cerebral vascular complications, whether they are thrombotic or hemorrhagic, remain rare, and only a handful number of cases have been reported^7^.

Cerebral venous sinus thrombosis (CVST) is a condition caused by multiple factors, and like any thrombotic process, risk factors are associated with classical Virchow triad: hypercoagulability, intimal injury, and blood stasis^8,9^. CVST may be caused by: tumors^10^, head trauma^11^, and central nervous system infections^8^.

Here, we report a rare case of a young patient that was diagnosed with neurobrucellosis complicated with CVST.

## Case presentation

A 16-year-old boy was admitted to the neurology ward, complaining of sudden onset of bilateral pulsatile headache that has started a week before admission. The headache was severe, refractory to over-the-counter painkillers, and accompanied by fever, photophobia, dizziness, nausea, vomiting, and blurred vision. The past medical history was unremarkable.

Upon admission his temperature was 38°C, blood pressure was 100/70 mmHg, heart rate was 100 bpm, respiratory rate was 20 breaths/ min. On physical examination the patient had neck stiffness, 2nd degree papilledema in both eyes, and the rest of examination was unrevealing.

Initial laboratory tests were as follows: WBC (9500/mm^3^) with a relative lymphocytosis, urea 17 mg/dl, creatinine 0.8 mg/dl, glucose 85 mg/dl, C-reactive protein (CRP) was 0.2 mg/dl (up to 0.5 mg/dl).

A brain computed tomography without contrast revealed no space-occupying lesions or any sign of hydrocephalus. Lumbar puncture showed: an opening pressure of 45 cmH_2_O, WBC (150/mm^3^) with a lymphocytic predominance (lymphocytes count 120 cell/mm^3^; 80%), RBC (20/mm^3^), glucose (52 mg/dl), and protein (39.7 mg/dl). The gram stain and culture were negative.

Based on these results, and since the patient was living in an endemic area for tuberculosis and Brucellosis, we tested the tuberculosis and Brucella PCR on the CSF sample. The Brucella PCR was positive, hence the diagnosis of neurobrucellosis was made.

**Figure 1:**
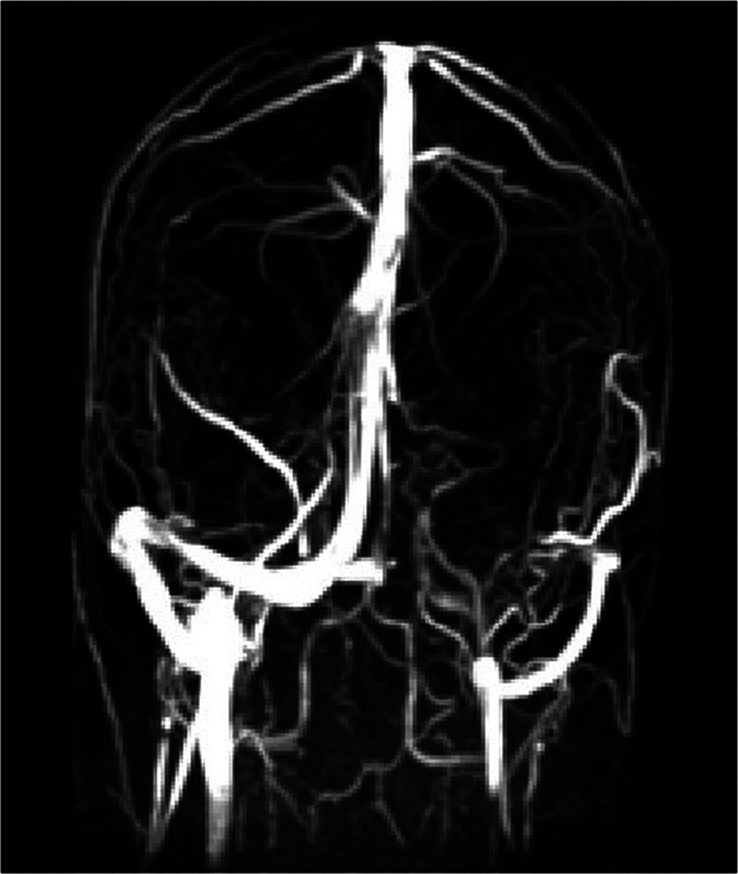
Magnetic resonance venography showing an absence of flow in the left transverse venous sinus.

**Figure 2:**
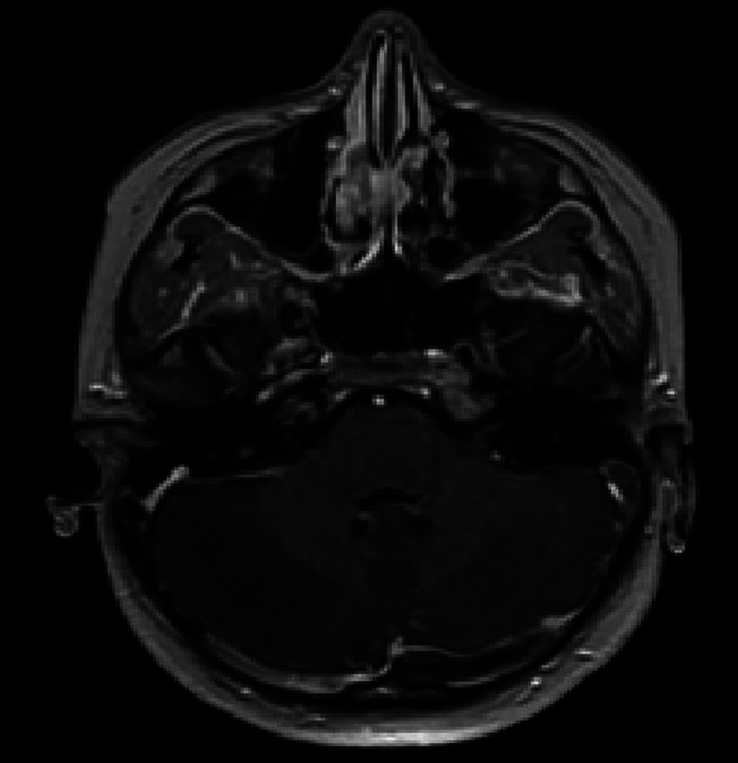
T1 with contrast showing a filling defect in the left transverse venous sinus.

Due to the elevated intracranial pressure, and the presence of papilledema, a brain MRI with contrast and magnetic resonance venography sequences was performed. The MRI findings were consistent with a left transverse venous thrombosis without leptomeningeal enhancement (Figs 1, 2).

These test results and imaging findings led to the diagnosis of neurobrucellosis complicated by CVST.

The patient was started on anticoagulation using low molecular weight heparin (LMWH); enoxaparin sodium 1 mg/kg/ subcutaneously (SC)/twice daily, and an antibiotic therapy (ceftriaxone 2 g/twice daily for 4 weeks, doxycycline 100 mg/twice daily, and rifampicin 300 mg/twice daily).

The clinical status was rapidly improved within days, in which headache, fever, and blurred vision were resolved. The patient was discharged on oral anticoagulants (rivaroxaban 20 mg/once daily) and oral antibiotics (Doxycycline 100mg/ twice daily, Rifampicin 300mg/ twice daily) for 6 months. However, the patient was lost on follow-up after that.

## Discussion

Brucellosis is still a major health problem in developing countries. This infection can affect both the central and peripheral nervous systems. Although neurological complications are rare, they are well diverse, making the diagnosis difficult. Diagnosis of neurobrucellosis is based on clinical history with CSF showing pleocytosis (>20/mm^3^), elevated protein content (>45 mg/dl), low CSF/plasma glucose rate (<0.50), and the isolation of Brucella from blood or CSF, or positive Brucellosis serology in CSF^12^.

Isolation of Brucella bacteria is the gold standard for the diagnosis. However, Brucella culture is time and resource consuming, taking several weeks to grow on culture medium. That is why, methods on the PCR are becoming very useful^13^. Although, PCR has a great potential, its sensitivity (50–100%) and specificity (60–98%) are variable^14^.

Vascular complications of brucellosis are rare and it usually affects arteries more than veins^15^. Certain vascular complications have been reported like deep vein thrombosis, however, CVST has seldomly been described^16^.

Overall CVST is a rare and uncommon disease^17^, although some studies have shown that otitis, mastoiditis, sinusitis, meningitis, and systemic infection can cause CVST; neurobrucellosis has rarely been reported as a related cause^18^.

To the best of our knowledge, only a few cases of neurobrucellosis complicated with CVST were reported in the literature^7^. Zaidan *et al*. reported the first case of CVST as a complication of neurobrucellosis.^19^ After that, Faraji *et al*.^20^ described a case of persistent seizures resulting from neurobrucellosis complicated by CVST.

Our patient presented with fever, headache and papilledema, and CSF results showed an elevation of WBC with a lymphocytic predominance. Also, the Brucella PCR on CSF was positive and the brain MRI showed a left transverse sinus thrombosis, leading to the diagnosis of neurobrucellosis complicated with CVST.

The guideline for the treatment of neurobrucellosis is not well-defined; however, most studies suggest that a combination of 2 or 3 antibiotics is essential. Some studies recommend the following antibiotics: doxycycline, rifampicin, trimethoprim-sulfamethoxazole, streptomycin, and/or ceftriaxone for a period of 4–9 months^21^. Whether the anticoagulation therapy is useful or not remains controversial, some studies suggest that it prevents the extension of thrombosis and recommend its use in all cases of CVST rather it was caused by sepsis or not^22^.

Using direct oral anticoagulants (DOACs) to treat the CVST was associated with similar clinical and radiographic outcomes compared to warfarin, but DOACs were safer^23^.

In our case, we used a combination of three antibiotics (ceftriaxone, rifampicin, and doxycycline). The anticoagulation was started with LMWH followed by DOAC (rivaroxaban) and continued for 3–6 months. The patient showed a great improvement within days after starting treatment, without any complications.

The role of thrombophilia in CVST is still not established, and ordering the thrombophilic profile was not cost-effective especially in the presence of a risk factor (such as central nervous system infection)^23,24^. Moreover, acute thrombosis can transiently affect the thrombophilic tests (reducing antithrombin levels)^25^, for that reason, we opted to not order the thrombophilic profile in our case as the patient has a precipitating factor (neurobrucellosis infection) and it would not affect the treatment plan.

## Conclusion

Although Brucella seldom affects the nervous system, with CVST being an extremely rare complication. Physicians should consider Brucella as the cause of CVST in endemic areas. In spite of the controversial evidence of anticoagulation in CVST caused by neuro infections, the use of DOAC played a role in our patient improvement, and should be considered as a part of septic CVST treatment in some cases. Future studies should be conducted to determine the exact duration and benefit of anticoagulation in such conditions.

## Ethical approval

Written informed consent was obtained from the father of patient for publication of this case report and accompanying images, in line with local ethical approval requirements and in accordance with the Helsinki Declaration.

## Consent

Written informed consent was obtained from the patient’s parents/legal guardian for publication and any accompanying images. A copy of the written consent is available for review by the Editor-in-Chief of this journal on request.

## Source of funding

Not applicable.

## Author contribution

A.A., O.A., and N.S.: wrote the manuscript, submitted the article, and patient follow-up; S.H.: literature search; M.A.: made article correction.

## Conflicts of interest disclosure

The authors declare that they have no conflicts of interest regarding this study. The author declares that it has not been published elsewhere and that it has not been submitted simultaneously for publication elsewhere.

## Research registration unique identifying number (UIN)

1. Registry used: NA.
2. Unique identifying number for registration: N/A.
3. Hyperlink to your specific registration: N/A.

## Guarantor

Omar Alsamarrai.

## Data availability statement

The data are available from the corresponding author upon reasonable request.

## Provenance and peer review

Not applicable.
